# Supplementary material for: NLRP1 restricts butyrate producing commensals to exacerbate inflammatory bowel disease
Source: Nat Commun. 2018 Sep 13;9:3728. doi: 10.1038/s41467-018-06125-0 (PMC6137172; doi:10.1038/s41467-018-06125-0)
Supplement: Supplementary file 1 — Supplementary Information [file 41467_2018_6125_MOESM1_ESM.pdf]

Supplementary Information for Tye et al:

NLRP1 restricts butyrate producing commensals to exacerbate inflammatory bowel disease.

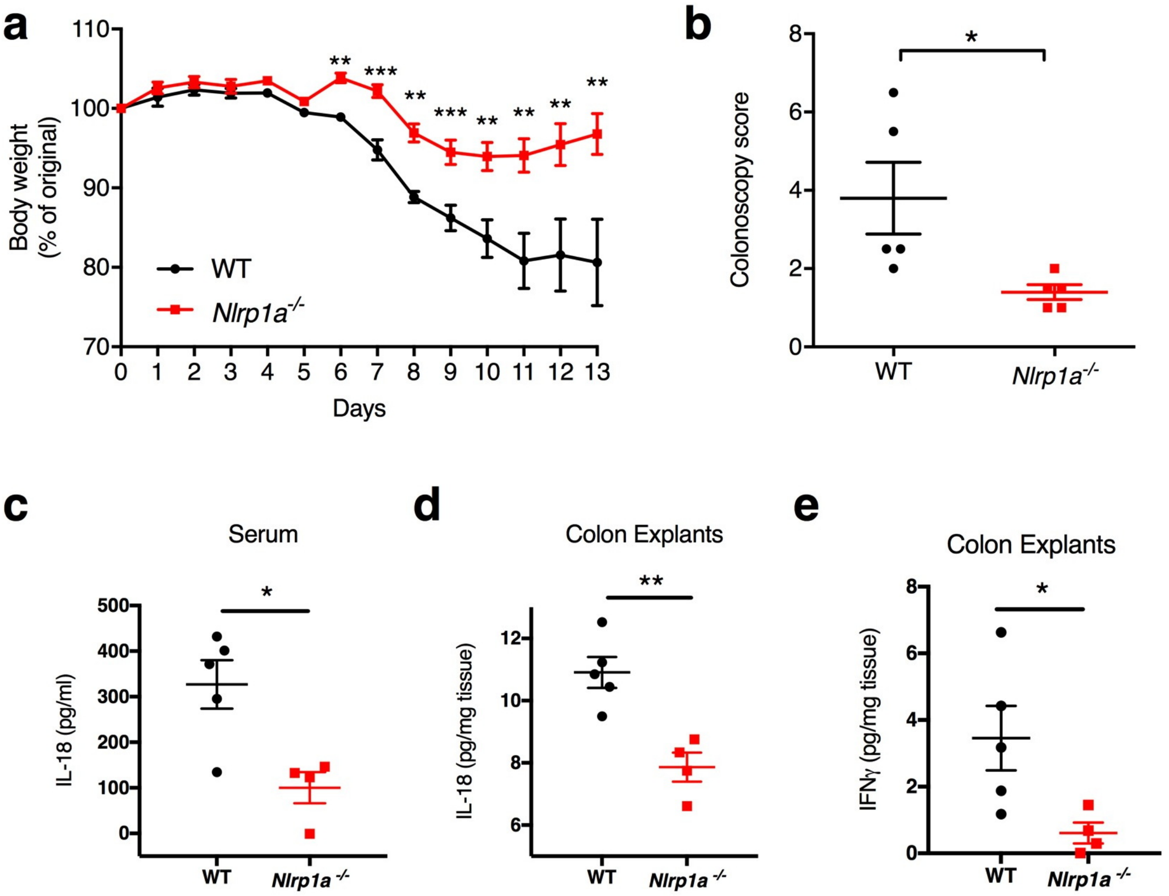

Supplementary Figure 1. Genetic deletion of Nlrp1a protects mice from DSS-induced colitis. **(a)** WT and *Nlrp1a*$^{-/-}$ mice were given 3% (w/v) DSS for 6 days followed by normal drinking water and monitored for weight loss. **(b)** Disease severity was measured by colonoscopy. **(c)** Serum was collected at day 7 and IL-18 quantified by ELISA. **(d)** Colon explants were cultured ex vivo and IL-18 or IFNγ quantified by ELISA. Data are representative of 2-3 independent experiments with 4-5 mice per group. Means ± SEM * p≤0.05, ** p≤0.01. As determined by a two-tailed, unpaired *t*-test.

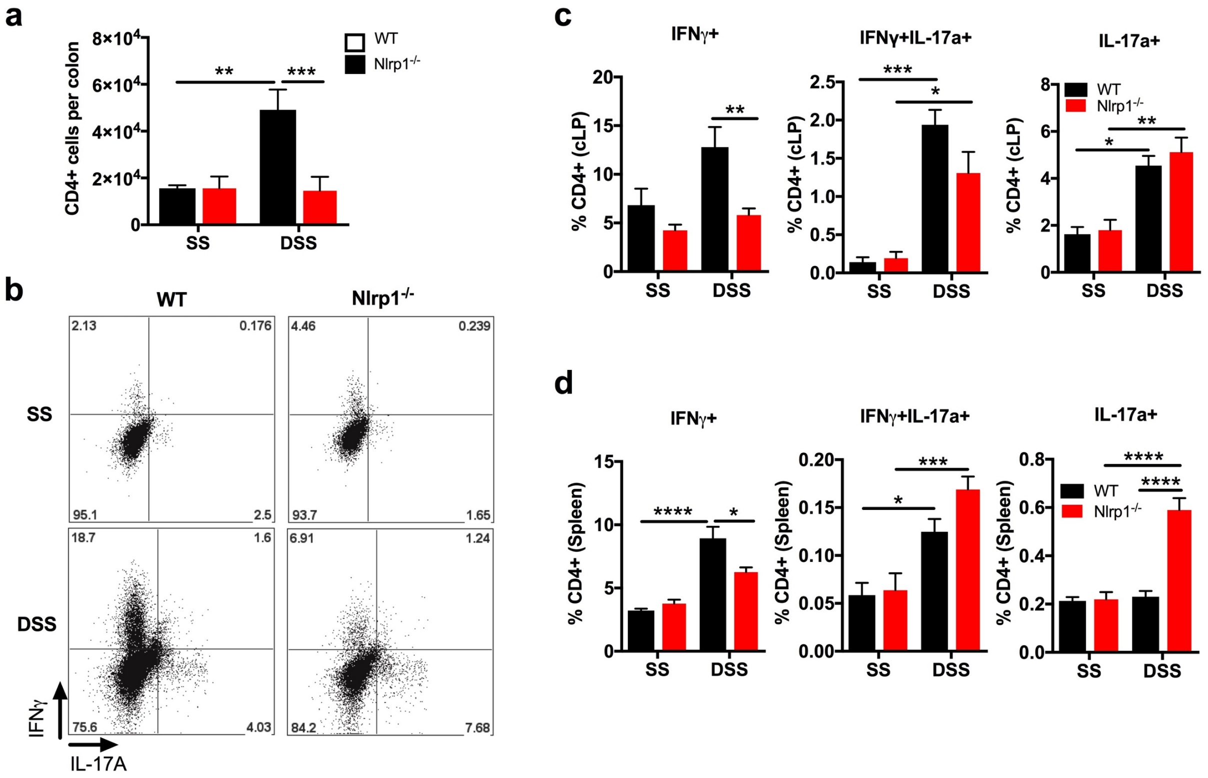

Supplementary Figure 2. The absence of NLRP1 during DSS-induced colitis is associated with reduced IFNγ-producing CD4+ T cells in the colonic lamina propria. Flow cytometric analysis of the colonic lamina propria (cLP) from WT and *Nlrp1*$^{-/-}$ mice at steady state (SS) and at day 8 following DSS treatment. **(a)** Enumeration of the absolute numbers of CD4+ T cells (gated on CD45+CD3+ T cells) in the whole colon. Lymphocytes were isolated and re-stimulated with PMA/Ionomycin in the presence of Brefeldin A for 4 hrs at 37°C to measure IFNγ and IL-17a production. **(b)** Representative FACS plots of CD4+ T cells producing IFNγ and IL-17a. **(c)** The frequencies of the indicated cytokines from CD4+ T cells isolated from the cLP and **(d)** spleen of WT and *Nlrp1*$^{-/-}$ mice at SS and during DSS. Data are representative of 2 independent experiments with 3-5 mice per group. Means ± SEM * p≤0.05, ** p≤0.01, ***p≤0.001. As determined by a two-tailed, unpaired *t*-test. A one-way ANOVA and Tukey's post-hoc comparisons were performed on data that involved more than 2 comparisons.

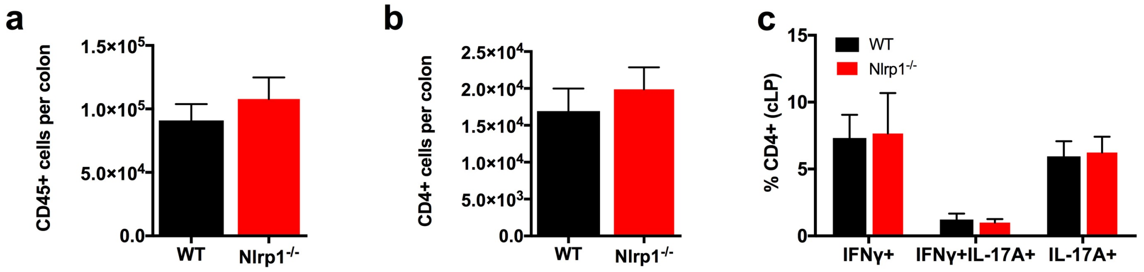

Supplementary Figure 3. Depletion of Gram-positive bacteria removes the IFNγ phenotype of Nlrp1$^{-/-}$ mice. Vancomycin treated WT and *Nlrp1*$^{-/-}$ mice at day 6 following DSS treatment were subjected to flow cytometric analysis of **(a)** CD45+ cells, **(b)** CD4+ T cells (gated on CD45+CD3+ T cells) and **(c)** PMA/Ionomycin re-stimulated lymphocytes for cytokine analysis of IFNγ and IL-17a production in the colonic lamina propria (cLP). Data are representative of 2 independent experiments with 3-5 mice per group. Means ± SEM. A two-tailed unpaired *t*-test was used to determine statistical significance between two groups; and a two-way ANOVA with Tukey's post-hoc comparisons were performed on data that involved more than 2 comparisons.

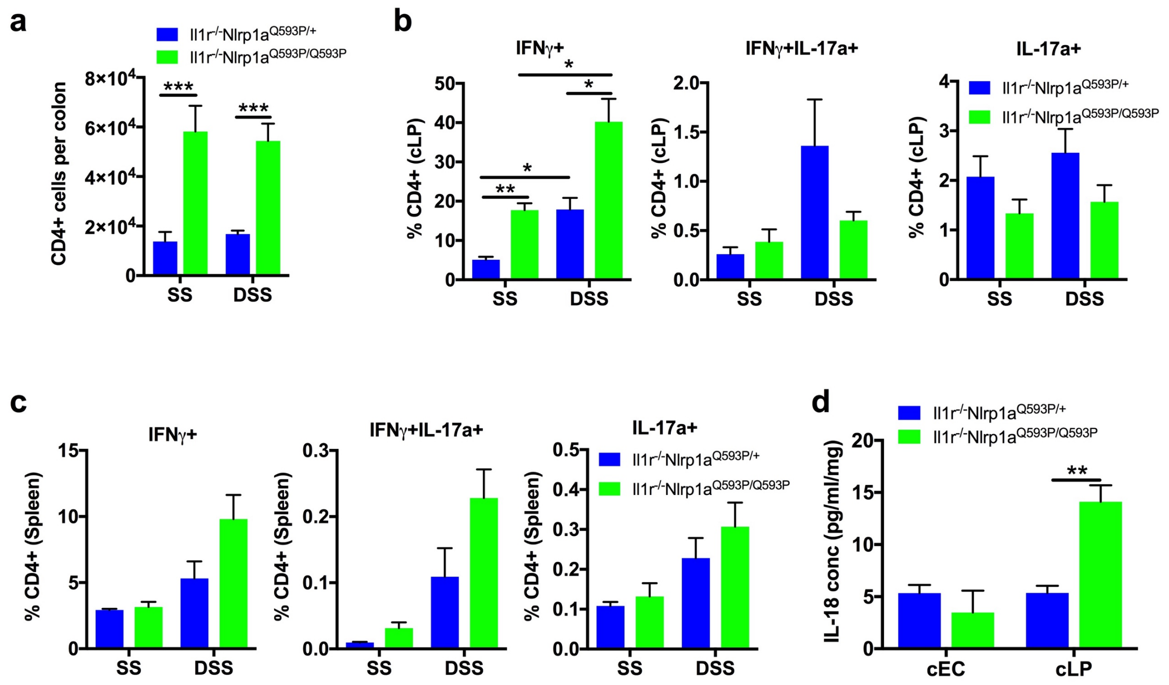

Supplementary Figure 4. NLRP1 activation mediates IFNγ producing CD4+ T cells in the cLP. (**a**) Enumeration of the absolute numbers of CD4+ T cells (gated on CD45+CD3+ T cells) in the whole colon. Lymphocytes from *Il-1r*$^{-/-}$*Nlrp1a*$^{Q593P/+}$ and *Il-1r*$^{-/-}$*Nlrp1a*$^{Q593P/Q593P}$ mice at SS or DSS-treated were re-stimulated with PMA/Ionomycin in the presence of Brefeldin A for 4 hrs at 37°C to measure IFNγ and IL-17a production from CD4+ T cells. The frequencies of the indicated cytokines from CD4+ T cells isolated from the (**b**) cLP and (**c**) spleen of *Il-1r*$^{-/-}$*Nlrp1a*$^{Q593P/+}$ and *Il-1r*$^{-/-}$*Nlrp1a*$^{Q593P/Q593P}$ mice at SS or during DSS-induced colitis. (**d**) IL-18 secretion from colonic epithelial cells (cEC) and the cLP cultures from *Il-1r*$^{-/-}$ *Nlrp1a*$^{Q593P/+}$ and *Il-1r*$^{-/-}$*Nlrp1a*$^{Q593P/Q593P}$ mice on DSS were measured by ELISA, normalized to colon weight. Data are representative of 2 independent experiments with 3-5 mice per group. Means ± SEM * p≤0.05, ** p≤0.01, ***p≤0.001, ****p≤0.0001. As determined by a two-tailed, unpaired *t*-test. A one-way ANOVA and Tukey's post-hoc comparisons were performed on data that involved more than 2 comparisons

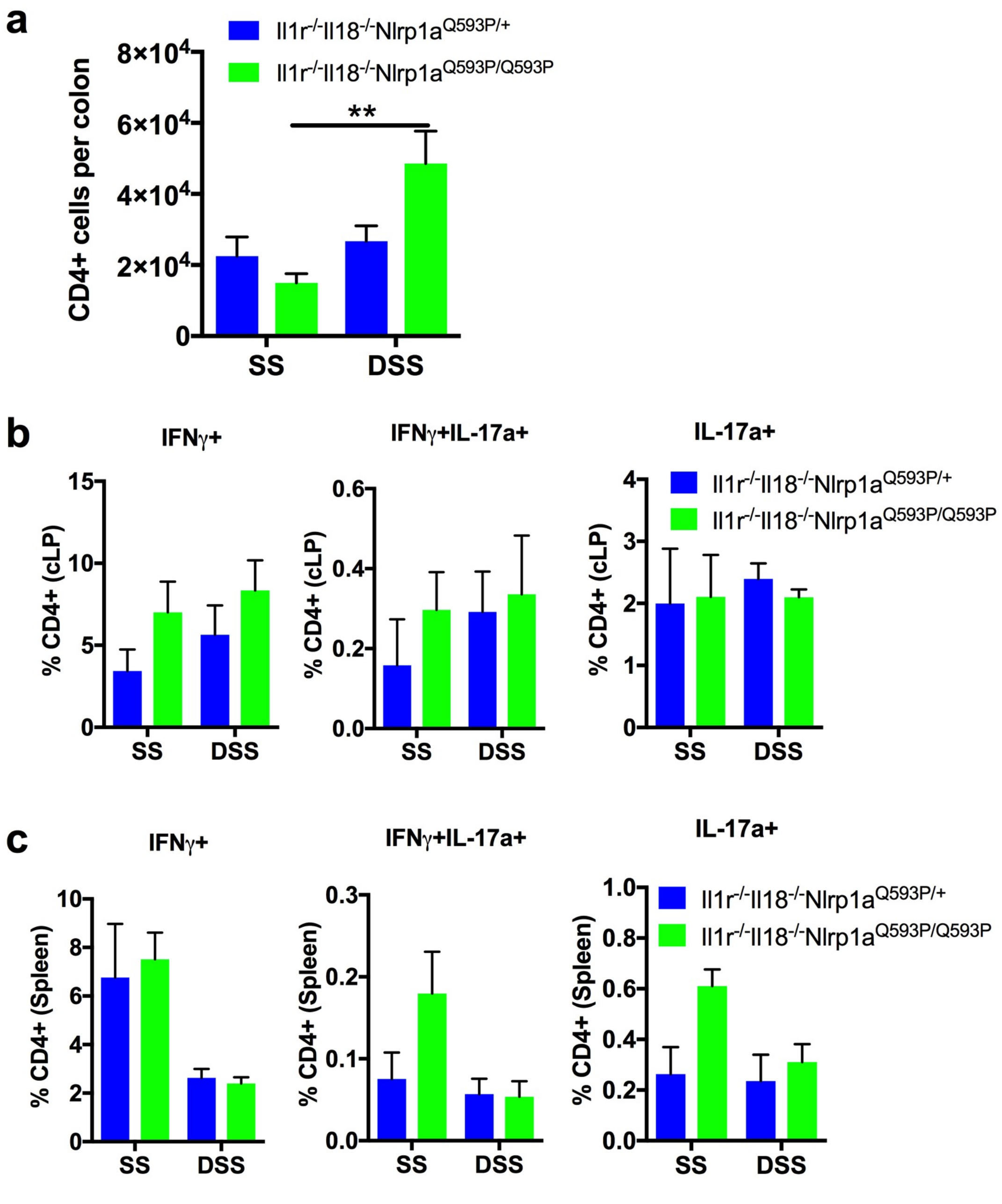

Supplementary Figure 5. Deletion of IL-18 removes the hyper-activated NLRP1 DSS-induced colitis phenotype. (**a**) Enumeration of the absolute numbers of CD4+ T cells (gated on CD45+CD3+ T cells) in the whole colon. Lymphocytes from *Il-1r*$^{-/-}$*Il-18*$^{-/-}$*Nlrp1a*$^{Q593P/+}$and*Il-1r*$^{-/-}$*Il-18*$^{-/-}$*Nlrp1a*$^{Q593P/Q593P}$mice at SS or DSS-treated were re-stimulated with PMA/Ionomycin in the presence of Brefeldin A for 4 hrs at 37°C to measure IFNγ and IL-17a production from CD4+ T cells. The frequencies of the indicated cytokines from CD4+ T cells isolated from the (**b**) cLP and (**c**) spleen of *Il-1r*$^{-/-}$*Il-18*$^{-/-}$*Nlrp1a*$^{Q593P/+}$and *Il-1r*$^{-/-}$*Il-18*$^{-/-}$*Nlrp1a*$^{Q593P/Q593P}$mice at SS and during DSS. Data are from 2 independent experiments with 2-4 mice per group. Means ± SEM * $p \leq 0.05$, ** $p \leq 0.01$, ***$p \leq 0.001$, ****$p \leq 0.0001$. As determined by a two-tailed, unpaired *t*-test. A one-way ANOVA and Tukey's post-hoc comparisons were performed on data that involved more than 2 comparisons

Supplementary Table 1. Pearson correlation of NLRP1 expression with OTUs from 16S sequencing of intestinal biopsies.

p-value	correlation coefficient	OTU	Order
0.007282	-0.38652	p__Firmicutes__g__Faecalibacterium__s__prausnitzii_366794	Clostridiales
0.020848	-0.33623	p__Firmicutes__f__Ruminococcaceae_308333	Clostridiales
0.026661	-0.32327	p__Actinobacteria__g__Rothia__s__mucilaginosa_1017181	Actinomycetales
0.032773	-0.31198	p__Firmicutes__f__Lachnospiraceae_New.ReferenceOTU215	Clostridiales
0.037669	-0.30414	p__Bacteroidetes__g__Bacteroides_3472078	Bacteroidales
0.045023	-0.2938	p__Bacteroidetes__g__Bacteroides_198449	Bacteroidales
0.046614	-0.29175	p__Firmicutes__g__Oscillospira_348009	Clostridiales
0.048138	-0.28983	p__Firmicutes__g__Veillonella__s__parvula_518743	Veillonellales

Supplementary Table 2. Pearson correlation of IL-18 expression with OTUs from 16S sequencing of intestinal biopsies.

p-value	correlation coefficient	OTU	Order
0.004094	-0.41114	p__Proteobacteria__f__Enterobacteriaceae_821080	Enterobacterales
0.008991	-0.37703	p__Firmicutes__g__.Eubacterium.__s__dolichum_42372	Clostridiales
0.013926	-0.35644	p__Firmicutes__o__Clostridiales_366986	Clostridiales
0.014411	-0.35477	p__Fusobacteria__g__Fusobacterium_298592	Fusobacteriales
0.019342	-0.34008	p__Firmicutes__g__WAL_1855D_1086889	Clostridiales
0.020268	-0.33768	p__Firmicutes__g__Coprococcus_470382	Clostridiales
0.022268	-0.33281	p__Bacteroidetes__g__Bacteroides_3426658	Bacteroidales
0.028469	-0.31973	p__Proteobacteria__g__Sutterella_1820513	Burkholderiales
0.037622	-0.30421	p__Firmicutes__g__Faecalibacterium__s__prausnitzii_350121	Clostridiales

Supplementary Table 3. Pearson correlation of IFNγ expression with OTUs from 16S sequencing of intestinal biopsies.

p-value	correlation coefficient	OTU	Order
0.005588	-0.39806	p__Firmicutes__f__Lachnospiraceae_349862	Clostridiales
0.008753	-0.37825	p__Firmicutes__f__Lachnospiraceae_564806	Clostridiales
0.012358	-0.36219	p__Firmicutes__f__Clostridiaceae_712677	Clostridiales
0.020229	-0.33778	p__Firmicutes__g__Veillonella__s__dispar_342427	Veillonellales
0.020998	-0.33586	p__Firmicutes__f__Lachnospiraceae_368462	Clostridiales
0.028022	-0.32058	p__Firmicutes__f__Lachnospiraceae_535601	Clostridiales
0.028228	-0.32019	p__Firmicutes__g__.Eubacterium.__s__dolichum_579851	Clostridiales
0.031253	-0.31461	p__Bacteroidetes__g__Bacteroides_198788	Bacteroidales
0.037753	-0.30401	p__Firmicutes__f__Ruminococcaceae_540862	Clostridiales
0.040663	-0.29975	p__Firmicutes__g__Oscillospira_304777	Clostridiales
0.042313	-0.29744	p__Firmicutes__o__Clostridiales_361727	Clostridiales
0.043347	-0.29603	p__Firmicutes__f__Peptostreptococcaceae_628226	Clostridiales
0.04414	-0.29497	p__Firmicutes__f__Erysipelotrichaceae_New.ReferenceOTU2677	Erysipelotrichales

Supplementary Table 4. Human subject characteristics.

Diagnosis	Healthy control (n=22)	Crohn's disease (n=33)	Ulcerative colitis (n=56)
Age (median, range (yr))	47.5 (28-60)	21 (9-42)	27 (8-72)
Gender (male)	10 (45%)	16 (49%)	23 (41%)
Ethnicity (Caucasian)	22 (100%)	33 (100%)	50 (89%)
Smoking			
ever	17 (85%)^	17 (52%)	22 (39%)
diagnosis		13 (39%)	8 (15%)*
biopsy	10 (50%)^	9 (27%)	2 (4%)*
Immunosuppression (ever) >3 months		25 (76%)	36 (64%)
Immunosuppression (biopsy) >3 months		12 (36%)	23 (41%)
Disease location (CD), biopsy			
L1 (ileal)		20	
L2 (colonic)		3	
L3 (ileocolonic)		10	
Disease behaviour (CD), ever			
B1 (inflammatory)		3	
B2 (stricturing)		19	
B3 (penetrating)		11	
Maximum disease extent (UC), biopsy*			
E1 (proctitis)			5
E2 (left-sided)			18
E3 (extensive)			32

*data was unavailable for one subject

^ data was unavailable for two subjects
